# Expression of VSTM1-v2 Is Increased in Peripheral Blood Mononuclear Cells from Patients with Rheumatoid Arthritis and Is Correlated with Disease Activity

**DOI:** 10.1371/journal.pone.0146805

**Published:** 2016-01-13

**Authors:** Dashan Wang, Yan Li, Yuan Liu, Yan He, Guixiu Shi

**Affiliations:** 1 Molecular Biology Research Center, Key Medical Health Laboratory for Laboratory Medicine of Shandong Province, Department of Laboratory Medicine, Shandong Medical College, Linyi, Shandong 276000, China; 2 Department of Rheumatology and Clinical Immunology, The First Affiliated Hospital of Xiamen University, Xiamen, Fujian 361003,China; University of Leuven, Rega Institute, BELGIUM

## Abstract

Rheumatoid arthritis (RA) is a chronic, systematic autoimmune disease that mainly affects joints and bones. Although the precise etiology is still unknown, Th17 cell is being recognized as an important mediator in pathogenesis of RA. VSTM1-v2 is a novel cytokine which has recently been reported to promote the differentiation of Th17 cells. This study is performed to study whether VSTM1-v2 can be recognized as a biomarker of RA, and is correlated to IL-17 expression. We obtained peripheral blood mononuclear cells (PBMCs) from 40 patients with RA and 40 age- and sex- matched healthy controls by standard Ficoll-Paque Plus density centrifugation. The mRNA expression levels of VSTM1-v2 and IL-17A in PBMCs were detected by real time-PCR. Disease activity parameters of RA were measured by routine methods. Our results showed that VSTM1-v2 mRNA expression in PBMCs from RA patients was significantly increased in comparison of that in healthy individuals. The VSTM1-v2 mRNA expression level was positively correlated with IL-17A mRNA expression level, DAS28, CRP and ESR, but was not correlated to RF, Anti-CCP or ANA. VSTM1-v2 might be a biomarker of RA and a novel factor in the pathogenesis of RA.

## Introduction

Rheumatoid arthritis (RA), a systematic autoimmune disease, is characterized by infiltration of synovium with immune cells leading to joint destruction [1]. Although the pathogenesis of RA is still unclear, many factors such as pro-inflammatory cytokines, immune cells, and genetic factors were considered to be implicated in pathogenesis RA [2]. The discovery of T helper 17 cells (Th17), which are characterized by secreting their signature cytokine IL-17A [3, 4], increased our understanding of the pathogenesis of RA. IL-17A can induce secretion of many pro-inflammatory cytokines from macrophage, and production of autoantibody from B cell[5, 6]. IL-17A can further promote cartilage destruction and bone erosion, besides, IL-17 receptor signaling pathway has been found critical to turn an acute synovitis into a chronic destructive arthritis [7, 8]. Th17 cells are considered to be a key factor in the initiation and maintenance of autoimmune arthritis [9, 10]. With the rapid development of genomics, proteomics and metabonomics, many different omics has emerged [11, 12]. Immunomics is one of the attractive areas. Immunomics is to study entire effectors which compose the mammalian immune system. Immunomics is relied on the use of large genomics, protein databases, and high throughput technology, to identify novel immune-related molecules and study there molecular mechanisms [13–15]. Based on immunomics strategy, many novel immune-related genes has been identified. V-set and transmembrane domain containing 1 (VSTM1) is one of them. Human VSTM1 is located on chromosome 19q13.42. There are two RNA splicing forms released in the NCBI nr database, VSTM1-v1 and VSTM1-v2. According to structural characteristics of cytokines, VSTM1-v2 may be a novel potential cytokine. A recent study showed that VSTM1-v2 is mainly expressed in immune tissues and cells, what is more, VSTM1-v2 can promote the differentiation and activation of Th17 cells [16]. Based on critical role of VSTM1-v2 in Th17 cell differentiation and the importance of Th17 cell in RA, VSTM1-v2 is probably involved in RA. However, whether VSTM1-v2 is associated with the development of RA has not been addressed yet. In the current study, we studied the expression of VSTM1-v2 mRNA in PBMCs from patients with RA, and analyzed the relationship between VSTM1-v2 and IL-17A. We further analyzed the relationship between VSTM1-v2 expression level and clinical characteristics of RA, including 28-joints disease activity score (DAS28), C-reactive protein (CRP), rheumatoid factor (RF), erythrocyte sedimentation rate (ESR), anti-cyclic citrullinated protein antibodies (anti-CCP) and antinuclear antibodies (ANA). Our results showed that the VSTM1-v2 mRNA expression was significantly increased in the PBMCs from RA patients compared with which in healthy controls. The IL-17A mRNA expression in PBMCs from RA patients was also significantly increased. The VSTM1-v2 mRNA expression level was positively correlated with the IL-17A mRNA expression level, DAS28, CRP and ESR, but is not correlated to RF, Anti-CCP or ANA. In conclusion, our results demonstrate that VSTM1-v2 expression is increased in PBMCs from RA patients and correlated to IL-17A expression and disease activity.

## Materials and Methods

### Patients and controls

A total of 40 preliminary diagnosed RA patients, who fulfilled the 1987 American College of Rheumatology revised criteria [17], were recruited from the outpatient clinic of the Department of Rheumatology, West China Hospital, Sichuan University. 40 sex-matched and age-matched healthy volunteers were recruited as controls. None of the patients had ever taken anti-rheumatic drugs, corticosteroids or vitamin D. Patients who had renal insufficiency or inflammatory disease were also excluded. The study was done after obtaining the approval of the Ethics Committee of West China Hospital and the written informed consent of the patients. RA disease activity was assessed using 28-joints disease activity score (DAS28), which has been widely used in the clinic to monitor disease activity of patients with rheumatoid arthritis [18, 19]. The demographic and clinical features of healthy controls and patients with RA were summarized in Table 1.

**Table 1:** Demographic and clinical characteristics of the patients with rheumatoid arthritis (RA) and healthy control subjects.

	RA patients (n = 40)	Healthy Controls (n = 40)
Age (years)	46.8 ± 11.2 ^Δ^	45.3 ± 9.16
Female (%)	85% ^Δ^	75%
C-reactive protein(mg/L)	30.6(1.1–158)	—
Rheumatoid factor (IU/ml)	326.0(6–1760)	—
DAS28* score	3.53(1.05–6.88)	—

*DAS28 = 28-joints disease activity score. Data are presented as mean (range) or mean ± standard deviation unless otherwise indicated.

^Δ^, *P* > 0.05, RA patients vs. healthy controls.

### Blood samples

Peripheral blood samples were collected into collection tubes containing 0.2ml sodium heparin from RA patients and healthy volunteers. Peripheral blood mononuclear cells (PBMCs) were isolated from heparinized blood by standard density-gradient centrifugation using Ficoll-Paque Plus (Axis-Shied PoC AS, Oslo, Norway).

### Reverse transcription—polymerase chain reaction (RT-PCR) and quantitative PCR

Total RNA was extracted from PBMCs by TRIzol^™^ Reagent (Invitrogen, Carlsbad, CA) and reverse-transcribed to cDNA with reverse transcription reagent kits according to manufacturer’s instructions (Bio-Rad, Hercules, CA, USA). The expression levels of VSTM1-v2, IL-17A, and GAPDH were determined by real-time quantitative PCR. The primer sequences were listed in S1 Table. A 10 μL SsoFast EvaGreen PCR reaction mixture was used containing 2μL of cDNA, 0.2μL of sense primer, 0.2μL of antisense primer, 2.6μL ddH2O, and 5μL Sso Fast EvaGreen Supermix (Bio-Rad, Hercules, CA, USA). Cycling conditions were as follows: 95°C for 1 min, followed by 40 cycles of 95°C for 10 s, 60°C for 10 s and 72°C for 10 s. Quantitative PCR was performed with the iQ^™^5 real time-PCR Detection Systems (Bio-Rad, Hercules, CA). The quality of RNA and the specificity and sensitivity of primers had been checked before performing gene detection. RNA, primers and PCR conditions all measure up to requirement. Gene expressions were normalized to those of GAPDH and relative expression was calculated by the 2^–ΔΔCt^ method.

### Statistical analysis

Data were analyzed with Prism 5.01 software (GraphPad Software, San Diego, CA) using Mann-Whitney test. The correlation between the VSTM1-v2 level and IL-17A level or clinical characteristics were analyzed using Spearman test. Data are shown as mean ± SD. *P* < 0.05 were considered significant.

## Results

### Increased VSTM1-v2 expression in PBMCs from patients with RA

**Fig 1:**
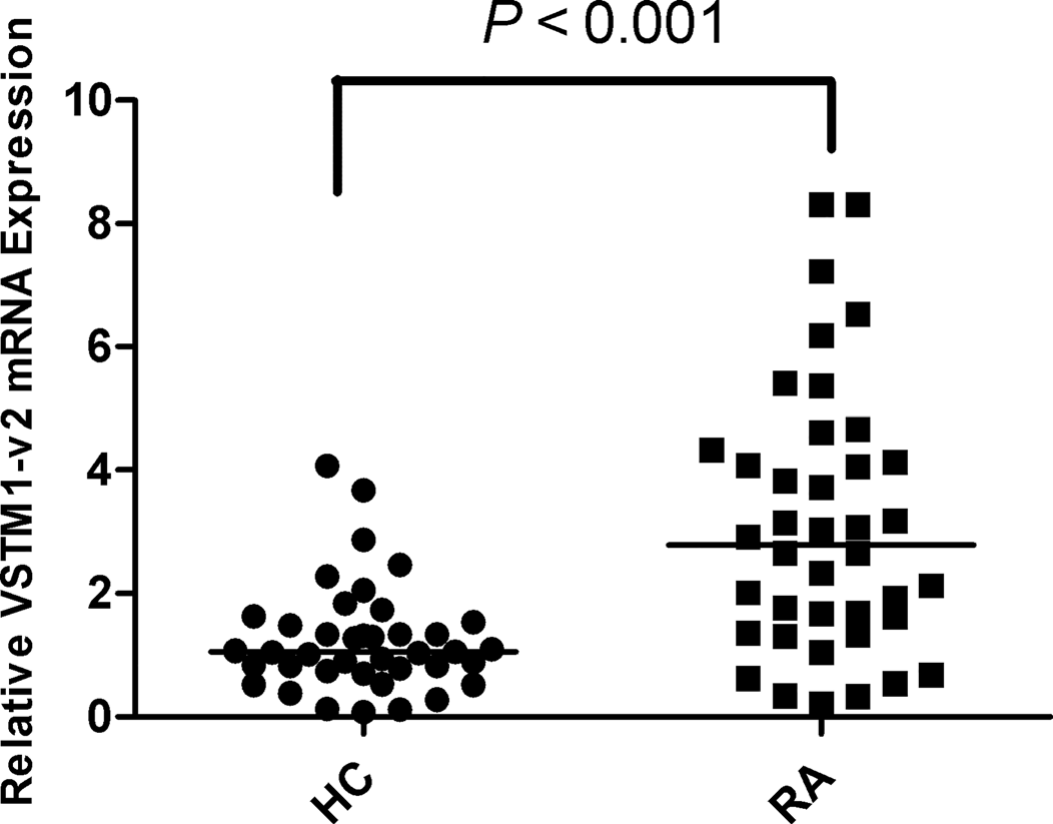
Increased VSTM1-v2 expression in PBMCs from RA patients. The expression of VSTM1-v2 mRNA was detected by real time-PCR. Relative VSTM1-v2 mRNA expression was normalized to GAPDH in PBMCs from patients with rheumatoid arthritis (RA; n = 40) and healthy controls (HC; n = 40). Data are shown as scatter plot. *P* value was determined by Mann-Whitney test.

According to the expressional and structural characteristics of cytokines, VSTM1-v2 may be a novel potential cytokine. To assess whether VSTM1-v2 plays a role in RA, we detected VSTM1-v2 mRNA expression in PBMCs from RA patients and healthy controls by real time-PCR. Results showed that mRNA expression of VSTM1-v2 was significantly higher in RA group than healthy control group (*p* < 0.001) (Fig 1).

### The relationship between the expression of VSTM1-v2 and IL-17A in patients with RA

**Fig 2:**
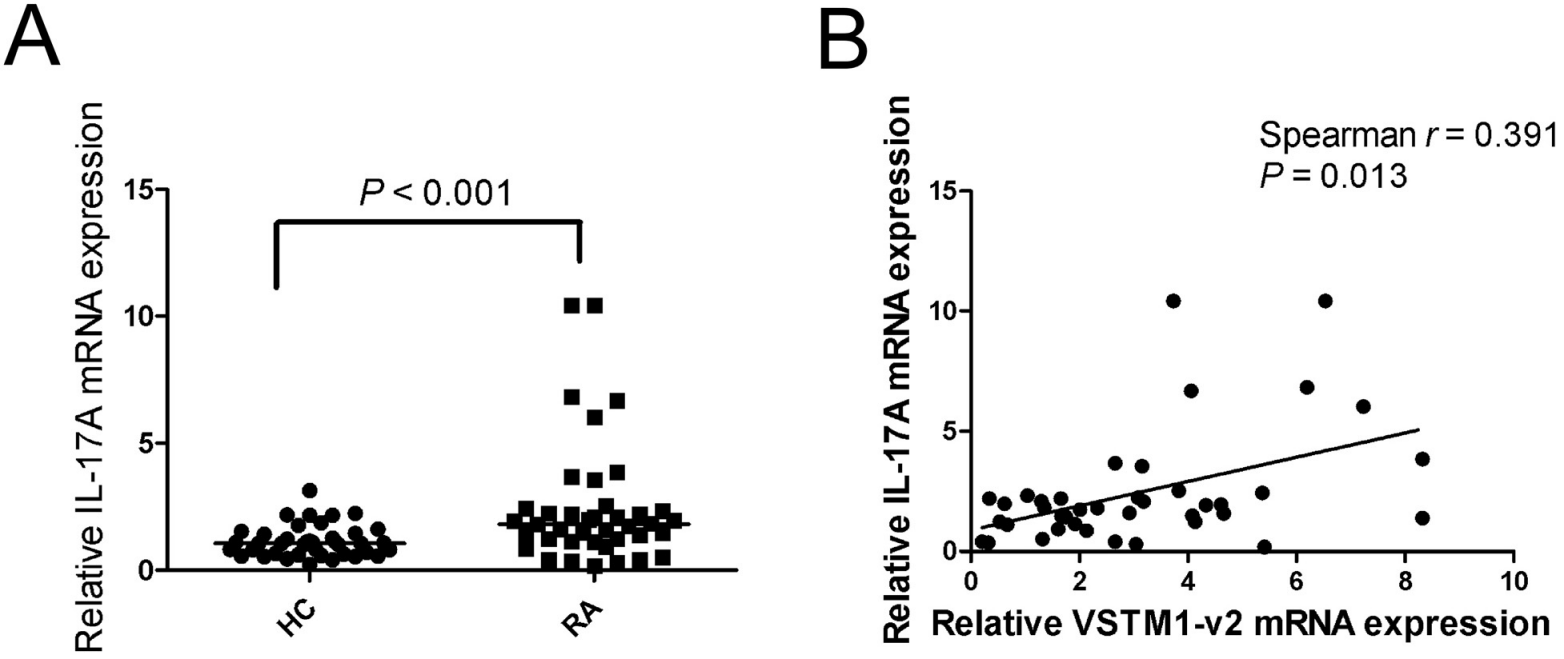
Correlation of VSTM1-v2 expression level and IL-17A mRNA expression level. (A) The expression IL-17A mRNA was detected with real time-PCR. Relative IL-17A mRNA was normalized to GAPDH in PBMCs from patients with rheumatoid arthritis (RA; n = 40) and healthy controls (HC; n = 40). Data are shown as scatter plot. *P* value was determined by Mann-Whitney test. (B) The correlation between VSTM1-v2 mRNA expression level and IL-17A mRNA expression level was determined by using Spearman test. The relation between VSTM1-v2 and IL-17A was presented.

Our data has suggested that VSTM1-v2 expression was increased in PBMCs from RA patients. As Th17 cells play a central role in pathogenesis of RA and VSTM1-v2 is involved in Th17 cell differentiation, we further detected RNA expression of IL-17A, and analyzed the relationship between expression of VSTM1-v2 and IL-17A in patients with RA. Consistent with previous report [20], our result showed IL-17A mRNA expression was significantly higher in RA group compared with healthy control group (*p* < 0.001) (Fig 2A). We also found a strong positive correlation between the VSTM1-v2 expression level and IL-17A expression level in patients with RA (*p* = 0.013) (Fig 2B).

### The correlation between VSTM1-v2 expression and RA disease characters

**Fig 3:**
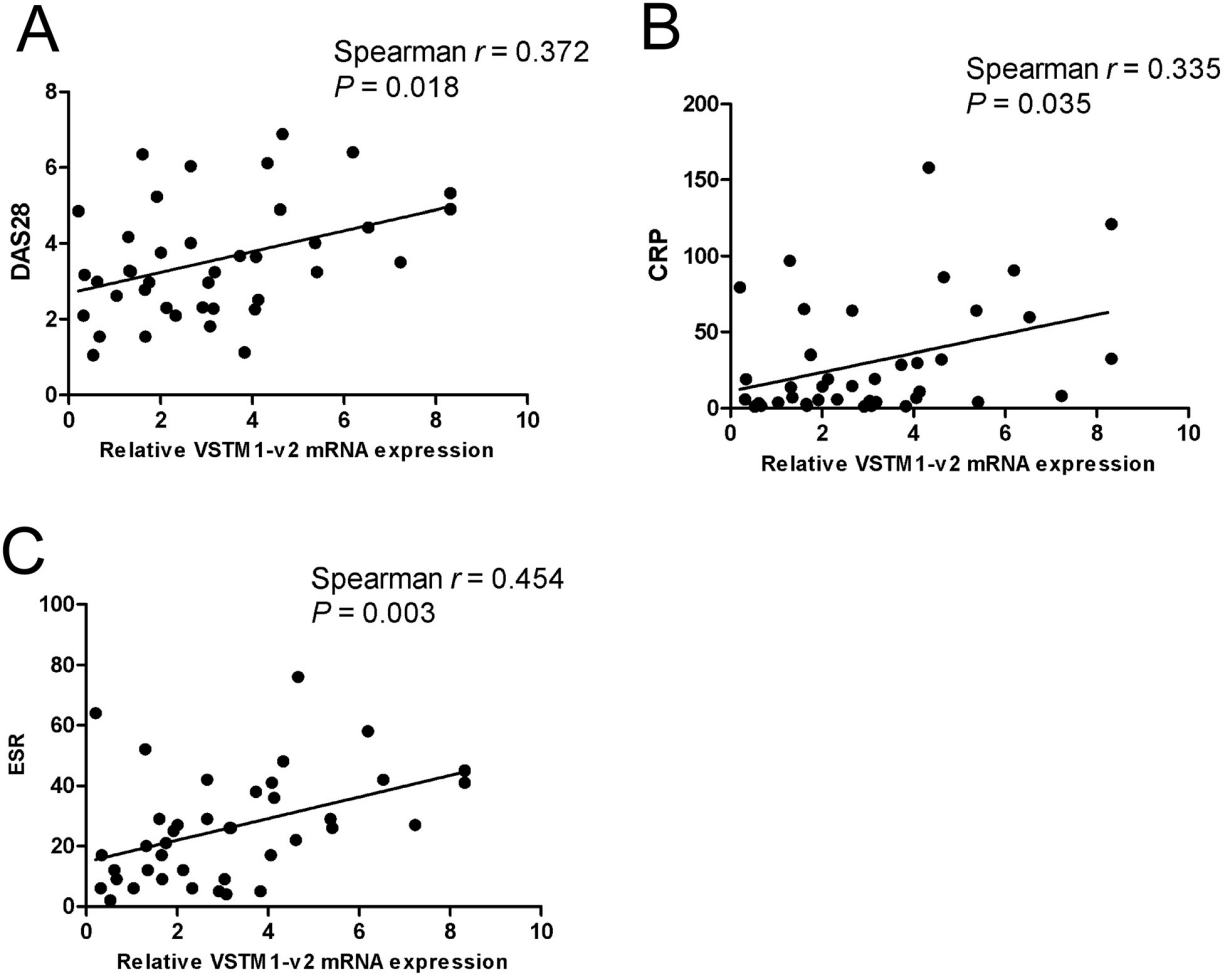
Expression of VSTM1-v2 in PBMCs from RA patients correlates with DAS28, CRP and ESR. The correlations between VSTM1-v2 mRNA expression level and RA disease characteristics were determined by using Spearman test. The correlations between VSTM1-v2 expression level and 28-joints disease activity score (DAS28) (Fig 3A), C-reactive protein (CRP) (Fig 3B), and erythrocyte sedimentation rate (ESR) (Fig 3C) were presented.

**Fig 4:**
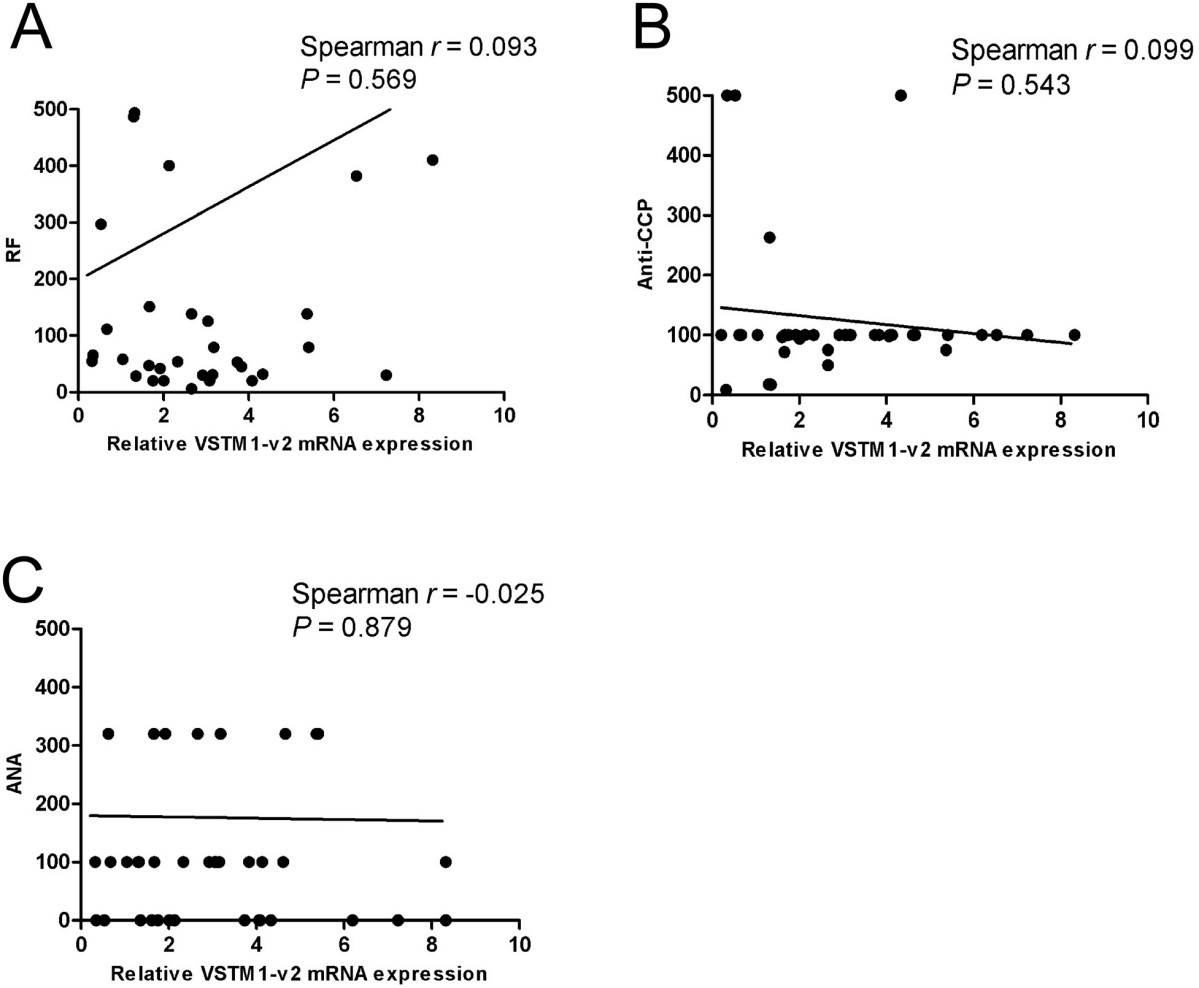
Correlations of VSTM1-v2 mRNA expression on PBMCs in RA patients with RF, Anti-CCP and ANA. The correlations between VSTM1-v2 mRNA expression level and RA disease characteristics were determined by using Spearman test. The correlations between VSTM1-v2 expression level and rheumatoid factor (RF) (Fig 4A), anti-cyclic citrullinated protein antibodies (anti-CCP) (Fig 4B) and antinuclear antibodies (ANA) (Fig 4C) were presented.

Our data have suggested that VSTM1-v2 expression was increased in PBMCs from patients with RA. To further study the association of VSTM1-v2 with human RA, we analyzed the relationship between VSTM1-v2 mRNA expression level and RA disease characters. We found strong positive correlations between the VSTM1-v2 expression level and DAS28 (*p* = 0.018), CRP (*p* = 0.035) or ESR (*p* = 0.003) (Fig 3). However, the VSTM1-v2 expression level was not correlated to RF level (*p* = 0.569), Anti-CCP (*p* = 0.543) or ANA (*p* = 0.879) in patients with RA (Fig 4).

## Discussion

In the present study, we showed that the mRNA expression of VSTM1-v2, a novel cytokine, was increased in patients with RA. The VSTM1-v2 expression level was positively correlated the expression level of IL-17A, which plays a central role in the pathogenesis of RA. Furthermore, the VSTM1-v2 expression level is positively related to RA disease characters. VSTM1-v2 may be a biomarker of RA and participate in the pathogenesis of RA.

Human *VSTM1* is located on chromosome 19q13.42. There are two RNA splicing forms, VSTM1-v1, a type I transmembrane protein, and VSTM1-v2, a classical secretory glycoprotein. According to bioinformatics, VSTM1-v1 may be an inhibitory receptor located on NK cells, VSTM1-v2 is a classical secretory protein[16]. By far, there are only a few papers on VSTM1-v2. One paper reported a new approach for selecting potential anti-VSTM1-v2 antibody[21] and another paper induced a system to product VSTM1-v2[22], the function of VSTM1-v2 is less studied. A recent study reported that VSTM1-v2 is mainly expressed in immune systems including bone marrow, thymus, lymph nodes, spleen and tonsil. VSTM1-v2 is in accordance with the structural and expressional characteristics of cytokines, thus, VSTM1-v2 seems to be considered as a novel potential cytokine. They further studied the function of VSTM1-v2, results showed that VSTM1-v2 can promote IL-17A secretion in CD4^+^ cells isolated from PBMCs, what is more, VSTM1-v2 can promote the differentiation of Th17 cells[16]. These results indicate that VSTM1-v2, a novel potential cytokine expressed in immune tissues, is closely related to differentiation and activation of Th17 cells. Rheumatoid arthritis (RA) is a complex, chronic systemic autoimmune disease characterized by joints or synovium inflammation, autoantibody production, cartilage and bone destruction. Although the precise etiology is still unknown, many recent studies suggest that Th17 cells play a central role in initiation and maintenance of RA. IL-17, a signature cytokine of Th17 cells, can lead to secretion of several other inflammatory factors such as tumor necrosis factor alpha (TNF-α), IL-1β that result in cartilage and joint ankylosis[23]. Previous studies showed that IL-17 expression in serum or synovial fluid of patients with RA is significantly higher than healthy controls and is associated with disease activity[24, 25]. As VSTM1-v2 regulates Th17 cell differentiation and Th17 cells play a central role in pathogenesis of RA, VSTM1-v2 might be associated with RA. In this study, we demonstrated that the VSTM1-v2 mRNA expression was correlated with IL-17A mRNA expression in patients with RA. These results indicate that VSTM1-v2 may be involved in the pathogenesis and progression of RA by controlling Th17 cell differentiation.

RA is commonly recognized as an autoimmune disorder that results from a complex interplay among genotype, environmental factors and also by chance[26]. Many RA risk gene has been identified in several large studies by the use of single nucleotide polymorphism (SNP) array, genome-wide association studies (GWAS), and immunochip (iCHIP)[27]. Among of them, major histocompatibility complex (MHC) locus variance has been repeatedly confirmed in RA patients. Besides, other non-MHC genes related to RA have also been identifying[28]. A research group aimed to identify novel functional variants in 398 candidated genes using targeted exon sequencing of RA in Korea. Although they did not find any novel variant with genome-wide significance for RA risk in a meta-analysis using a Korean RA GWAS, iCHIP data and their results, however in a gene-based analysis using nonburden tests (optimal sequence kernel association test (SKATO)) and burden tests (SCORE-seq) *VSTM1* showed the greatest association with RA. Seven nonsynonymous variants of *VSTM1* were identified. This result indicated that variants of *VSTM1* is probable associated with RA in Korean RA patients[29]. Our result showed that VSTM1-v2 mRNA expression level was significantly increased in patients with RA in comparing to health control. Perhaps the change of VSTM1-v2 expression is attributed to mutations of *VSTM1*. However whether variants of VSTM1 are linked to RA in Chinese population need further study.

There are some limitations of this study. First, as mentioned above in the last paragraph, we found the expression of VSTM1-v2 is significantly increased in patients with RA, but we didn’t address the reason for increased VSTM1-v2 expression. Whether the increased VSTM1-v2 expression is attributed to SNP, DNA methylation, histone acetylation or other causes is worth further studying. Additionally, we included 40 RA patients and 40 healthy controls in our study, increased VSTM1-v2 expression and its correlation between some clinical characteristics in RA were identified in current study, these results can become more convincing by enlarging sample size.

## Conclusions

Taken together, we report that VSTM1-v2 expression is increased in PBMCs of patients with RA, and the VSTM1-v2 expression level is correlated with IL-17A expression level and RA disease activity. VSTM1-v2 might be a biomarker of RA and a novel factor in the pathogenesis of RA.

## Supporting Information

S1 Table Primer sequences used for quantitative real-time PCR analysis. (DOC)
