# Supplementary material for: Expression of VSTM1-v2 Is Increased in Peripheral Blood Mononuclear Cells from Patients with Rheumatoid Arthritis and Is Correlated with Disease Activity
Source: PLoS One. 2016 Jan 13;11(1):e0146805. doi: 10.1371/journal.pone.0146805 (PMC4711949; doi:10.1371/journal.pone.0146805)
Supplement: S1 Table — (DOC) [file pone.0146805.s001.doc]

**S1 Table**. Primer sequences used for quantitative real-time PCR analysis.

| **Gene Name** | **Forward Primer Sequence** | **Reverse Primer Sequence** |
| --- | --- | --- |
| VSTM1-v2 | 5’-CAGCCATTCCAAACTTCC-3’ | 5’-CACTTTCAGTGCCGCATA-3’ |
| IL-17A | 5’- CAACCGATCCACCTCACCTT-3’ | 5’-GGCACTTTGCCTCCCAGAT-3’ |
| GAPDH | 5’-GTGAACCATGAGAAGTATGACAAC-3’ | 5’-CATGAGTCCTTCCACGATACC-3’ |

VSTM1-v2, V-set and transmembrane domain containing 1-v2; IL-17A, Interleukin-17A; GAPDH, glyceraldehyde-3-phosphate dehydrogenase
